# STRESSOR CHARACTERIZATION IN STUDIES OF PHYSICAL RESILIENCE

**DOI:** 10.1093/geroni/igad104.0376

**Published:** 2023-12-21

**Authors:** Karen Bandeen-Roche, Qianli Xue, Fangyu Liu, Ravi Varadhan, Brian Buta, Charles Brown IV, Jeremy Walston, Frederick Sieber

**Affiliations:** Johns Hopkins University, Baltimore, Maryland, United States; Johns Hopkins, Baltimore, Maryland, United States; Johns Hopkins Bloomberg School of Public Health, Baltimore, Maryland, United States; Johns Hopkins University School of Medicine, Baltimore, Maryland, United States; Johns Hopkins University, Baltimore, Maryland, United States; School of Medicine, Baltimore, Maryland, United States; Johns Hopkins University, Baltimore, Maryland, United States; Johns Hopkins University School of Medicine, Baltimore, Maryland, United States

## Abstract

Physical resilience may be considered as rebounding well from physical stressors. Early gerontological consensus has emphasized that resilience properly should be referred to as “resiliencies”—each, specific to a given stressor type and magnitude. Specificities include that one may be resilient to a minor stressor but not a more severe one as well as that one’s physiologic state and constellation relevant to determining resilience capacity (“PRC”) may better accommodate certain types of stressors than others. Yet, the literature on resilience in older adults has devoted little attention to stressor characterization. In this talk, a conceptual framework for considering the role of stressor variation in studies of resiliency and a process to elicit expert opinion on sources of variation in stressor experience are presented. Both are applied both within the RESiliency in TOtal knee REplacement (RESTORE) study. The conceptual framework distinguishes “exogenous” (potentially randomizable) and “endogenous” (connected to one’s PRC) variation in stressor magnitude and type. Elicitation identified anesthesia aspects and surgical technique elements among exogenous features, and perioperative vital indicators, bleeding and pain amelioration among endogenous features. Stressor feature variation was analyzed: this was found to be highest for perioperative vasopressor use, body temperature and postoperative opioid requirements. Analyses describing feature associations with resilience outcomes also will be presented: These examine the validation hypothesis that more severe stressors are associated with worse outcomes. This work is producing measures of stressor magnitude and type suitable for testing the primary RESTORE hypothesis that fitter PRC blunts adverse stressor effects on outcomes.

